# Real-time kinetic studies of *Mycobacterium tuberculosis* LexA–DNA interaction

**DOI:** 10.1042/BSR20211419

**Published:** 2021-11-18

**Authors:** Chitral Chatterjee, Soneya Majumdar, Sachin Deshpande, Deepak Pant, Saravanan Matheshwaran

**Affiliations:** 1 Department of Biological Sciences and Bioengineering, Indian Institute of Technology, Kanpur, U.P., India; 2 Centre for Environmental Sciences and Engineering, Indian Institute of Technology Kanpur, U.P., India

**Keywords:** “SOS” response, Bio-layer Interferometry, DNA binding kinetics, EMSA, LexA, Mycobacterium tuberculosis

## Abstract

Transcriptional repressor, LexA, regulates the ‘SOS’ response, an indispensable bacterial DNA damage repair machinery. Compared with its *Escherichia coli* ortholog, LexA from *Mycobacterium tuberculosis* (Mtb) possesses a unique N-terminal extension of additional 24 amino acids in its DNA-binding domain (DBD) and 18 amino acids insertion at its hinge region that connects the DBD to the C-terminal dimerization/autoproteolysis domain. Despite the importance of LexA in ‘SOS’ regulation, Mtb LexA remains poorly characterized and the functional importance of its additional amino acids remained elusive. In addition, the lack of data on kinetic parameters of Mtb LexA–DNA interaction prompted us to perform kinetic analyses of Mtb LexA and its deletion variants using Bio-layer Interferometry (BLI). Mtb LexA is seen to bind to different ‘SOS’ boxes, DNA sequences present in the operator regions of damage-inducible genes, with comparable nanomolar affinity. Deletion of 18 amino acids from the linker region is found to affect DNA binding unlike the deletion of the N-terminal stretch of extra 24 amino acids. The conserved RKG motif has been found to be critical for DNA binding. Overall, the present study provides insights into the kinetics of the interaction between Mtb LexA and its target ‘SOS’ boxes. The kinetic parameters obtained for DNA binding of Mtb LexA would be instrumental to clearly understand the mechanism of ‘SOS’ regulation and activation in Mtb.

## Introduction

The expression of DNA damage and stress response genes, which serve to preserve genome integrity upon exposure to DNA damaging agents, is controlled by the ‘SOS’ response pathway. Activation of the ‘SOS’ response helps the bacteria to develop resistance to antibiotics, making it indispensable for survival and growth under adverse conditions [1–3]. This pathway is regulated by two key players, namely, RecA and LexA. LexA binds to a consensus sequence of DNA known as the ‘SOS’ box located in the operator region of several genes and transcriptionally represses them under normal physiological conditions. However, under stress conditions, LexA falls off from the operators leading to activation of these genes to facilitate DNA repair [4]. The ‘SOS’ regulons exhibit significant variations across the bacterial kingdom, reflecting their overall complexity. For example, while *Bacillus subtilis* harbors only 33 genes in its ‘SOS’ regulon, *Escherichia coli* contains over 45 genes [5]. Most of the ‘SOS’ regulons include genes that encode for error-prone DNA polymerases, LexA, RecA, and proteins involved in the nucleotide excision repair pathway, although exceptions are known to exist [6].

‘SOS’ activation occurs in the following sequence of events—(i) RecA interacts with single-stranded DNA to form activated nucleoprotein filament complex, (ii) activated RecA directly interacts with LexA leading to autoproteolytic cleavage of the latter, and finally (iii) LexA falls off from the operator regions causing transcriptional de-repression of the damage-inducible genes [7]. In *E. coli*, the LexA repressor binds to consensus ‘SOS’ box sequence present in different operators, with variable affinity [7]. Genes with lower operator-repressor affinity are activated early on in the ‘SOS’ pathway when compared with genes having tightly bound operators. For instance, genes such as *lexA, uvrA, uvrB, uvrD*, and *recA* express early on, while those encoding for error-prone polymerases, (*dnaE2, umuD*) and *sulA* express much later in the cascade [7]. Altogether, the differential binding of LexA to different operators results in a highly complex but well-coordinated process that protects the cellular machinery during DNA damage.

LexA homologs are widespread across bacterial genomes and have remained evolutionarily conserved [8]. While most of the DNA damage-inducible genes in *E. coli* are under the direct control of LexA/RecA, such is not in the case of *Mycobacterium tuberculosis* (Mtb), wherein several DNA damage-inducible genes are independent of LexA regulation [9]. Among the 21 genes regulated by LexA/RecA in Mtb, only a few have been characterized, limiting our present understanding of the molecular mechanisms underlying the regulatory role of LexA. The functional characterization of LexA, including estimation of kinetic parameters obtained from binding to different ‘SOS’ boxes, will provide new insights in mechanistically understanding mycobacterial ‘SOS’ regulation.

Well-studied LexA homologs from *E. coli* and *B. subtilis* are known to exhibit nanomolar binding affinities toward ‘SOS’ box DNA with an apparent *K*_D_ of 0.8 and 2.3 nM, respectively [10,11]. However, a lack of kinetic analyses for Mtb LexA–DNA interaction greatly limits our understanding of mycobacterial ‘SOS’ activation. Nearly 25 *in-*binding sites of LexA were identified in the Mtb genome by Davis and colleagues in 2012 [12]. The study demonstrates differential expression of the damage-inducible genes upon Mitomycin C-induced stress [12]. Precise quantitation of such molecular interactions of Mtb LexA with DNA could reveal the importance of structural integrity and the mechanism of ‘SOS’ induction. To address this, it is imperative to study the kinetics of the interaction of LexA with its target binding sites and thus we carried out real-time Mtb LexA–DNA interaction studies.

Although the C-terminal domain (CTD) crystal structure of Mtb LexA provides a molecular framework for its architecture and assembly, the DNA-binding domain (DBD) remained inaccessible for structural studies [13]. Mtb LexA has an N-terminal DBD and a C-terminal dimerization/catalytic domain separated by a linker region similar to its orthologs from other bacteria. Notably, sequence comparison between Mtb and *E. coli* LexA revealed a unique N-terminal extension of 24 amino acids, which came into light after the re-annotation of Mtb LexA by Smollett and colleagues in 2009 [14], and a longer linker region with additional 18 amino acids in Mtb LexA. Further, the functional importance of these extra stretches of amino acids in and around the DBD and linker region of Mtb LexA remains unclear. Therefore, in the present study, we investigated whether these unique regions play any role in DNA binding. We have generated Mtb LexA mutants that lack 24 amino acids from its N-terminal domain (NTD), 18 amino acids from its linker region, in addition to mutating the conserved DNA binding residues ‘RKG’ to ‘AAA’. We have compared the DNA binding property of these mutants with wild-type Mtb LexA qualitatively using EMSA and quantitatively by Bio-layer Interferometry (BLI) to obtain real-time binding kinetic parameters. The DNA binding assessed using BLI revealed a comparable binding affinity of wild-type Mtb LexA and its N-terminal 24 amino acids truncation variant. Deletion of 18-amino acid from the linker resulted in 16 times reduced binding, whereas RKG mutant showed no binding, suggesting their importance in DNA binding. The kinetic parameters of Mtb LexA–DNA interaction obtained from the present study provide new functional insights crucial for an understanding of mycobacterial ‘SOS’ regulation.

## Materials and methods

### Plasmids, strains, and reagents

Bacterial strains and plasmids used in the study are listed in Supplementary Table S1. Primers and oligonucleotides were purchased from Sigma Aldrich and are listed along with the constructs generated in the study in Supplementary Table S2. All the reagents, media, and chemicals were purchased from Sigma Aldrich, Hi-Media, and SRL, and enzymes were purchased from New England Biolabs.

### Construction and analysis of phylogenetic tree

Multiple sequence alignment of LexA proteins from diverse bacterial groups was created using Clustal X, and the phylogenetic tree was constructed using RaxML [15]. The evolutionary history of the taxa has been inferred from 1000 replicates of the bootstrap consensus tree. The percentage of replicates in which clustering of related taxa took place is mentioned next to the branches. Two hundred and eighty-three positions were found in the final dataset. iTOL was used to visualize the generated tree [16].

### Over-expression and purification of proteins

The Mtb LexA and its ∆24aa variant were individually cloned between NdeI and BamHI restriction sites, respectively, in pET28a(+) bearing N-terminal 6× His tag. Mtb LexAΔ18aa and Mtb LexA RKG/AAA mutants were generated by the overlap PCR method. The primers used have been listed in Supplementary Table S2. All constructs generated have been confirmed by sequencing.

The recombinant WT and its variant proteins were over-expressed in *E. coli* BL21 (DE3) cells. Cultures were induced with 0.5 mM IPTG at OD_600_ 0.6. The cells were pelleted after 4 h by centrifugation at 5000×***g*** following which the pellet was resuspended in Lysis Buffer composed of 50 mM Tris-Cl (pH 8.0), 150 mM NaCl and 10 mM imidazole (pH 8.0), 5% glycerol, 1 mM phenylmethylsulfonyl fluoride (PMSF). After lysing the cells by sonication on ice (10” On, 30” Off cycles), the clarified lysate was centrifuged for 1 h at 20,000×***g***. The clarified supernatant was passed through a pre-equilibrated HisTrap HP column at 5 ml/min. Column was washed with 50 mM Tris-Cl, 500 mM NaCl, 30 mM imidazole (pH 8.0), and proteins were subsequently eluted under a gradient in 25 mM Tris-Cl (pH 8.0), 150 mM NaCl and 750 mM imidazole (pH 8.0). Pure fractions were pooled, diluted with a low salt buffer (25 mM Tris-Cl [pH 8], 100 mM NaCl, 1 mM EDTA, 5% glycerol), and loaded on to Q-Sepharose column for anion exchange chromatography. Proteins were eluted in gradient by passing a high salt buffer (25 mM Tris-Cl [pH 8.0], 1 M NaCl, and 1 mM EDTA). The pure fractions were concentrated using Gel Filtration Buffer (20 mM Tris-Cl [pH 8.0], 100 mM NaCl, and 5% glycerol) and separated using Superdex 75 10/300 GL for gel filtration. The purified proteins were run on 12% SDS-PAGE for analyzing their purity and concentrations were determined via spectrophotometric analysis.

### Cross-linking reactions

The cross-linking reactions were performed by incubation of each of the proteins at 5 µM final concentration in presence of 0.01% v/v glutaraldehyde in 10 mM HEPES (pH 8.0), 50 mM NaCl for 30 min on ice. The reactions were stopped with 25 mM of DTT. Samples were separated on a 12% SDS-PAGE.

### Circular dichroism

Circular dichroism (CD) spectra were recorded from 195 to 280 nm using a Jasco J-815 spectropolarimeter. A 1 mm pathlength quartz cuvette was used. Resolution up to 0.2 nm was maintained with a scan rate of 100 nm/min. The temperature of 25°C was maintained for all experiments. About 5 μM of each protein in 10 mM Tris-Cl 50 mM NaCl (pH 7.5) was taken for analysis. The data presented are an average of three scans after correction for the buffer baseline. Recorded spectra were analyzed using Origin 8.1 software.

### Extrinsic fluorescence

Extrinsic fluorescence spectra were obtained using a Jobin-Yvon Fluorometer FluoroMax3 at 25°C. About 5 μM of each of the proteins in 10 mM Tris-Cl (pH 7.5), 50 mM NaCl was incubated with 44 mer ds *dnaE2* ‘SOS’ box containing DNA (sequence given in Table 1) at 1:2 ratio for 30 min at 37°C. The samples were incubated with 40 μM of ANS in dark for 10 min. Samples were excited at 350 nm, and in the range of 400–600 nm, emission spectra were recorded. Measurements were corrected for fluorescence intensity of buffer, DNA, and ANS intrinsic fluorescence.

**Table 1:** ‘SOS’ boxes chosen for binding studies

Duplex	Oligonucleotides used for DNA binding analysis and kinetics^*^	
*dnaE2*_44mer	FP- 5′	Btn**^#^**-ACAACTGCGCTGTA**TCGAACAATTGTTCGA**TATACTGTGGAATG
	3′ RP- 3′	TGTTGACGCGACAT**AGCTTGTTAACAAGCT**ATATGACACCTTAC 5′
	(Perfect palindrome)	
*lexA*_44mer	FP- 5′	Btn-CCGGAACACGCCTG**TCGAACACATGTTTGA**TTCTTGGTGCGAAT 3′
	RP- 3′	GGCCTTGTGCGGAC**AGCTTGTGTACAAACT**AAGAACCACGCTTA 5′
	(**Imperfect** palindrome)	
*recA*_44mer	FP- 5′	Btn-GTGTCACACTTGAA**TCGAACAGGTGTTCGG**CTACTGTGGTGATC 3′
	RP- 3′	CACAGTGTGAACTT**AGCTTGTCCACAAGCC**GATGACACCACTAG 5′
	(**Imperfect** palindrome)	
*rv3074*_44mer	FP- 5′	Btn-GCAGGCTGCTATTC**TCGAACACATGTTCGA**GACATTGACCGCGA 3′
	RP- 3′	CGTCCGACGATAAG**AGCTTGTGTACAAGCT**CTGTAACTGGCGCT 5′
	(Perfect palindrome on the repeat flanks, not in the sequences **in between**)	

* ‘SOS’ box sequences are highlighted in **bold.**

# Btn stands for biotinylation.

### Electrophoretic mobility shift assay

WT and its variants at 128 nM were incubated in the presence of 3.5 nM of end-labeled (non-biotinylated) 44 mer ds *dnaE2* ‘SOS’ box containing DNA (sequence given in Table 1) in 10 mM HEPES (pH 7.5), 50 mM NaCl for 30 min on ice. The unbound DNA and DNA–protein complexes were resolved on 8% native PAGE at 100 V for 1 h in cold. Gels were dried and autoradiographed. The same procedure was followed for EMSA analysis in which increasing concentrations (0–128 nM) of WT Mtb LexA was incubated with ^32^P end-labeled ds 44 mer ds *dnaE2* ‘SOS’ box containing DNA.

### Bio-layer Interferometry (BLI)

The ForteBio Octet RED 96 (Forte Bio, U.S.A.) platform was used to conduct interaction studies between LexA and its variants with biotinylated ds 44mer of different ‘SOS’ boxes containing sequences (listed in Table 1). Streptavidin matrix-coated sensor chip (SA) was equilibrated in 10 mM HEPES (pH 7.5), 50 mM NaCl followed by immobilization of 100 nM of biotinylated ds DNA on it. Increasing concentrations of WT and mutant proteins were passed on to the chip and change in response units (RU) was analyzed. The program comprises 1 min stabilization of the baseline with the buffer followed by 10-min loading of sensors with biotinylated DNA, a 5-min association enabling interaction between the protein and DNA, a 5 min dissociation step finally followed by a 5 s regeneration step (unless mentioned otherwise). A reference sensor dipped in the buffer was used as a background control. All analyses were carried out at 25°C. A 1:1 binding model was applied to globally fit the binding isotherms and kinetic parameters such as *k*_on_, *k*_off_, and *K*_D_ were obtained. The experiments were performed in triplicates.

## Results and discussion

### Mtb LexA features distinct characteristics from its counterparts

LexA is present in most bacterial species and phyla [17]. Evolutionarily, the protein has retained its two distinct domains, the NTD, involved in DNA binding, and CTD, which is responsible for dimerization and autoproteolytic cleavage. Alpha helices involved in DNA binding and the residues critical for autoproteolysis have remained well conserved across different species, thereby preserving the overall functions of the protein.

**Figure 1:**
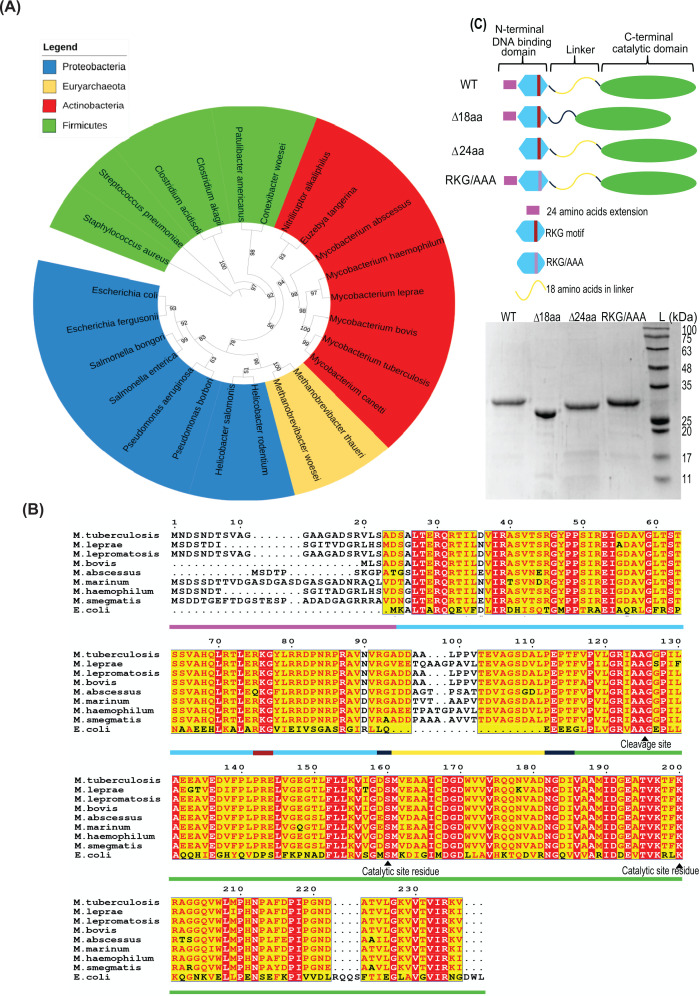
Phylogenetic analysis and domain architecture of Mtb LexA (**A**) Phylogenetic tree of LexA protein sequence of 24 species. The protein sequences are clustered into four groups: Actinobacteria, Proteobacteria, Euryarchaeota, and Firmicutes that include the representatives from some of the major groups of bacteria. (**B**) Comparison of LexA sequences among tuberculous, non-tuberculous mycobacterial species, and model Gram-negative organism, *E. coli*. The first 24 amino acids extension, the N-terminal DNA binding domain, linker region, the stretch of 18 amino acids insert in the linker, and C-terminal dimerization domain are shown as a bar representation below the sequence in magenta, light blue, dark blue, yellow, and light green, respectively. DNA-binding residues chosen for mutation are shown with a brown bar representation below. Sequence alignment was done using Clustal Omega, and ESPript was used to generate the Figure. (**C**) Representation of constructs generated for this study is shown, and the gel picture shows purified Mtb LexA and its variants resolved and visualized on 12% SDS PAGE.

From an evolutionary viewpoint, a comparison between LexA homologs from selected representatives belonging to major classes of Gram-positive, Gram-negative, Archaebacterial, and Actinobacterial phyla reveal the discrete clustering based on their classification, evident from the phylogenetic tree constructed (Figure 1A). This tree has been deduced by comparing sequences from 24 representative bacterial species (shown in Supplementary Figure S1). LexA homologs from Actinobacteria closely resemble homologs from Gram-positive Firmicutes which is consistent with their relatedness at the species level. Interestingly, LexA from members of Actinobacteria that include the pathogenic tuberculous mycobacteria such as Mtb, *M. canetti*, and* M. bovis* shows significant similarity, suggesting a possible link between pathogenicity and sequence evolution of LexA.

Subsequent comparison of the LexA sequences among some of the well-known tuberculous and non-tuberculous mycobacteria revealed interesting results. Although the C-terminal regions remain almost identical, N-terminal regions exhibit sequence variations especially at the terminal end. Pathogenic mycobacteria have relatively smaller genome sizes [18] and are not expected to code for unwanted additional stretches of amino acids in their proteins unless they prove advantageous for their survival. Interestingly, tuberculous mycobacteria except for *M. bovis* were found to possess additional amino acids at the N-terminal end of LexA, unlike their *E. coli* counterpart, implying unexplored adaptive functions. Another region of less conservation spans the linker region that connects the NTD and CTD of the protein. While the latter half of the linker sequences (toward the CTD) exhibits more conservation, the initial half varies among different mycobacterial species. Although the linker is identical between Mtb and *M. bovis* harboring 25 amino acids, the number of residues and sequence conservation vary among the other mycobacteria (Figure 1B). In *M. leprae* and *M. haemophilum*, the linker can extend up to 28 amino acids long. LexA possessing additional stretches of amino acids triggers the curiosity to explore their mechanistic roles in ‘SOS’ induction that could help mycobacterial survival and evolution.

Presumably, these stretches of amino acids in Mtb LexA that remain uncharacterized may confer additional functions unique to mycobacterial species when compared with its well-characterized homologs. LexA is a global repressor controlling the expression of DNA repair genes. Hence, assessing whether these additional stretches of amino acids may influence interactions with ‘SOS’ boxes could provide new information related to ‘SOS’ regulation. Considering both the additional stretches of amino acids to lie in proximity to the DNA binding regions of the protein, we deleted these stretches to explore their impact on DNA binding. Subsequently, the stretch of 24 amino acids (residues 1–24) was deleted to generate LexAΔ24aa, and LexAΔ18aa was generated by deleting 18 amino acids spanning the hinge region (residues 94–111) of the protein. This long hinge region separates the NTD from its CTD in Mtb LexA. This is in sharp contrast with the much shorter hinge region of just four amino acids (Q70-E74) present in *E. coli* (Figure 1B). The functional relevance of such a long hinge region in Mtb has not been explained. Possibly, the longer length of this inter-domain linker in Mtb LexA can enhance its flexibility to attain suitable conformations for binding to DNA [13]. Next, we have attempted to shed light on this aspect in the present study by characterizing and comparing the DNA-binding ability of the variants with that of WT LexA. RKG motif involved in DNA binding contains Arg 52, Lys 53, and Gly 54 in *E. coli* LexA lying in the third alpha helix of the protein [19], and its corresponding Arg 75, Lys 76, and Gly 77 residues of Mtb LexA have remained conserved. We have mutated these residues to assess whether they are essential for DNA binding in Mtb LexA.

### Evaluation of Mtb LexA and its variants for dimerization and DNA-binding property

**Figure 2:**
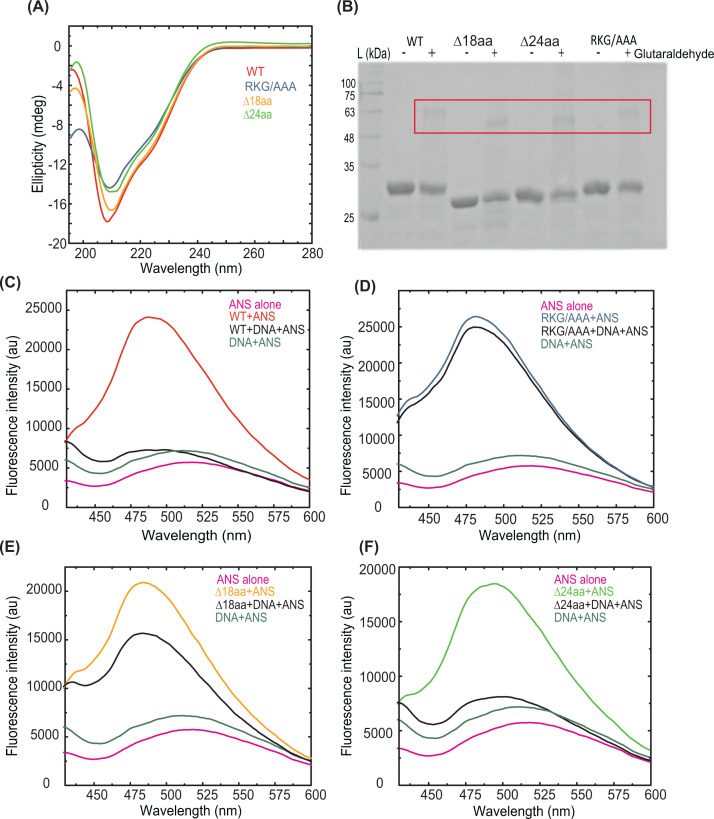
Biochemical analysis and DNA binding property of Mtb LexA and its variants (**A**) The secondary structure of WT Mtb LexA, LexAΔ24aa, LexAΔ18aa, and LexA RKG/AAA were compared using CD spectroscopy, monitored at wavelengths ranging from 195 to 280 nm. (**B**) Gel image showing glutaraldehyde cross-linking of Mtb LexA and its variants. Dimeric states of proteins are boxed. (**C-F**) Changes in extrinsic fluorescence spectra of the proteins, (C) WT Mtb LexA, (D) LexA RKG/AAA, (E) LexAΔ18aa and (F) LexAΔ24aa, as seen when incubated at 1:2 ratios with non-biotinylated *dnaE2* ‘SOS’ box (sequence given in Table 1) indicates the conformational changes of the proteins upon DNA binding. Fluorescence intensity is shown in arbitrary units.

All proteins were purified to ∼98% purity (Figure 1C). Near and far UV spectra of Mtb LexA and its mutants were used to assess changes in their secondary structures due to mutations (Figure 2A). Interestingly, both far (195–250 nm) and near (250–280 nm) UV spectra from CD studies revealed the comparable secondary structures of WT Mtb LexA and its mutants.

All the variants predominantly exist as dimers in solution as analyzed from profiles of gel filtration chromatography (Supplementary Figure S2). To confirm this further, the purified proteins were subjected to cross-linking using the chemical cross-linker glutaraldehyde. Upon cross-linking, the predominant form appeared to be dimeric in all cases as evident from the top band running between 48 and 63 kDa (Figure 2B). Structural analysis of the C-terminal segment of Mtb LexA revealed residues 229–236 along with residues 139–153 from its NTD to be involved in dimer formation between two LexA monomers [13]. The mutants generated in the present study have no overlapping sequences with the aforementioned residues and therefore, all of them retained the ability to form dimers. Therefore, a comparable profile of results in CD spectroscopy and protein dimerization experiments revealed that the selected regions had minimal or no influence on the secondary structure of Mtb LexA.

**Figure 3:**
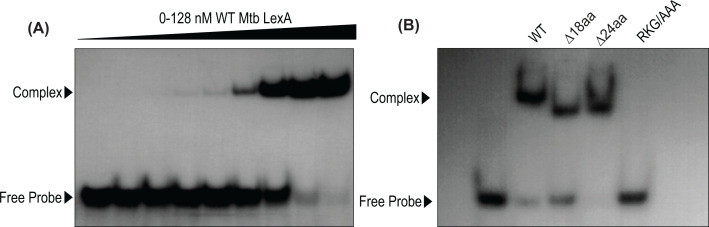
DNA binding properties of Mtb LexA and its variants compared by EMSA analysis (**A**) EMSA analysis of WT Mtb LexA binding to ^32^P end-labeled ds 44 mer ds *dnaE2* ‘SOS’ box containing DNA at 0, 1, 2, 4, 8, 16, 32, 64, and 128 nM is shown. (**B**) 128 nM of WT, LexAΔ24aa, LexAΔ18aa, and LexA RKG/AAA were incubated with 3.5 nM of ^32^P end-labeled ds 44 mer ds *dnaE2* ‘SOS’ box containing DNA (sequence given in Table 1), and EMSA was carried out according to standardized conditions mentioned in Materials and Methods.

Next, we used fluorescence spectroscopy to assess the structural changes of Mtb LexA and its variants upon interaction with DNA. Hydrophobic extrinsic fluorescent dye 8-anilino-1-naphthalenesulfonic acid (ANS) has been widely used for screening the alterations in the tertiary structure of proteins and to monitor their ligand-binding events such as protein–nucleic acid interactions [20]. The ligand displaces the fluorescent dye upon binding to the protein, resulting in a quench in fluorescence. Changes in fluorescence intensity are a direct readout of protein–DNA binding. The maximum quench in fluorescence intensity was noted when WT LexA formed a complex with DNA (Figure 2C). A similar quench in fluorescence intensity was observed for LexAΔ24aa and the WT protein upon DNA binding (Figure 2F), suggesting that the 24 amino acids extension is not crucial for DNA binding. However, we cannot overlook the possibility that it may have a regulatory role in DNA binding that could be dependent on the ‘SOS’ box sequences [21]. In striking contrast with LexAΔ24aa, deleting 18 amino acids from the linker region has significantly reduced the fluorescence quenching to nearly half compared with that for the WT, suggesting that this linker region may play a prominent role in DNA binding (Figure 2E). The deletion of 18 amino acids had weak or no effect on the secondary structures; however, the observed reduction in nucleic acid binding affinity may be influenced by the Van der Waals interactions offered by either glutamic acid or aspartic acid residues (4 out of 18 amino acid residues) within the linker region. Lastly, RKG/AAA mutant showed no significant fluorescence quench upon interaction with DNA (Figure 2D), thereby establishing that RKG residues play a critical role in DNA binding in Mtb LexA similar to the other orthologs. Further, we have carried out electrophoretic mobility shift assays (EMSA) to see the DNA binding of WT LexA to the *dnaE2* ‘SOS’ box. The shift in the ^32^P-labeled *dnaE2* ‘SOS’ box DNA suggests that Mtb LexA can bind to ‘SOS’ box with nM affinity (Figure 3A). We have also performed EMSA with Mtb LexA variants. EMSA analysis revealed that LexAΔ24aa and LexAΔ18aa showed mobility shift, whereas RKG/AAA mutant did not show any DNA binding (Figure 3B). This further confirms the importance of the RKG motif in DNA binding. Based on these observations, we further decided to quantitate the real-time kinetic parameters of LexA–DNA interactions with WT LexA and its variants.

### Determination of DNA binding kinetics of Mtb LexA and its variants

We used BLI for determining the DNA binding affinity for Mtb LexA and its mutants. First, we characterized the interaction between WT Mtb LexA and its variants to the perfectly palindromic *dnaE2* ‘SOS’ box (‘SOS’ box sequence given in Table 1). WT and its variant, LexAΔ24aa, exhibit comparable DNA binding affinity (*K*_D_ of 2.16 ± 0.01 nM for WT and 4.94 ± 0.03 nM for LexAΔ24aa, respectively). Further, LexAΔ24aa with and without N-terminal 6x His tag showed comparable DNA binding kinetics (Supplementary Figure S3).

**Figure 4:**
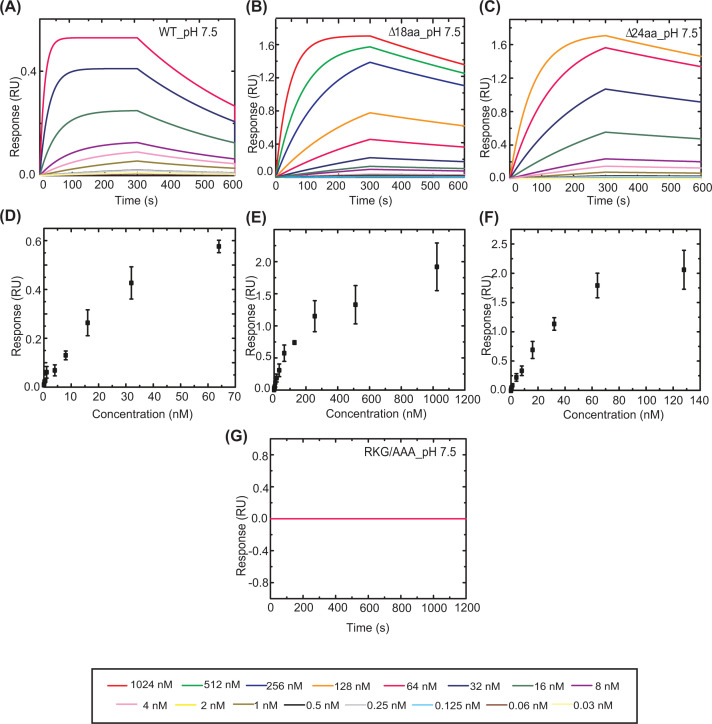
Kinetic analysis of DNA binding of Mtb LexA and its variants to *dnaE2* ‘SOS’ box at physiological pH Representative BLI sensograms showing the real-time concentration-dependent (each concentration depicted by a different color indicated in the box below) binding of WT Mtb LexA and its variants, LexAΔ18aa, and LexAΔ24aa to biotinylated 44 mer *dnaE2* ‘SOS’ box at physiological pH (**A–C**), the kinetic parameters obtained from which are tabulated in Table 2. The corresponding response versus concentration curves has been plotted in (**D**–**F**) from the results of three independent experiments. (**G**) Representative BLI sensogram showing absence of binding of LexA RKG/AAA to biotinylated 44 mer *dnaE2* ‘SOS’ box at physiological pH.

LexA18aaΔ variant shows significantly reduced affinity as seen from the sensograms depicted in Figure 4. Deleting 18 amino acids from the linker connecting NTD and CTD resulted in a more than 15 times reduction in affinity when compared with the full-length protein, with an obtained *K*_D_ of 34.4 ± 0.19 nM (Table 2). The rate of association (*k*_on_) was highest in the order of 10^6^ for full-length Mtb LexA and decreased to the order of 10^4^ in the case of the ∆18aa variant. While the association rate constants varied, the dissociation rate constants (*k*_off_) did not change significantly. We speculate that deleting the 18 amino acids from the linker affected the conformation to bind DNA suitably, thereby reducing its association rate (Figure 4B and Table 2). We did not observe any detectable binding for RKG/AAA, even after doubling the time of interaction (from 300 to 600 s of association) (Figure 4G), indicating the RKG motif in DBDs is critical for the DNA binding. Altogether, Mtb LexA exhibits similarity to *E. coli* counterpart in terms of binding to its cognate ‘SOS’ box with nanomolar affinity. Additionally, the presence of the longer linker in Mtb LexA is found to positively affect its DNA-binding ability. The kinetic parameters obtained by performing experiments at physiological pH intrigued us to investigate how they would vary when subjected to acidic conditions. The rationale behind choosing a highly acidic pH condition to monitor changes in DNA binding affinity of Mtb LexA arises from the fact that Mtb is known to face a hostile environment of acidic pH inside host macrophages and is challenged to maintain internal pH homeostasis for survival [22]. Although internal pH lower than 6 is noted to be lethal for mycobacteria, it has to endure external pH as low as 4 [22]. We wanted to assess whether Mtb LexA and its variants could exhibit DNA binding even at an extreme pH such as pH 4.

**Table 2:** Kinetic parameters obtained from binding studies performed using BLI

Protein	*k*_on_ (M^−1^s^−1^) × 10^5^	*k*_off_ (1/s) (s^−1^) × 10^−3^	*K*_D_ (nM)	*R* ^2^
LexA (pH 7.5)	10.55 ± 0.06	2.28 ± 0.007	2.16 ± 0.01	0.99
LexA∆18aa (pH 7.5)	0.22 ± 0.0007	0.76 ± 0.004	34.4 ± 0.19	0.99
LexA∆24aa (pH 7.5)	1.06 ± 0.003	0.52 ± 0.003	4.94 ± 0.03	0.99
LexA (pH 4)	0.20 ± 0.0006	1.52 ± 0.002	75.99 ± 0.27	0.99
LexA∆18aa (pH 4)	0.15 ± 0.0004	1.64 ± 0.002	111 ± 0.33	0.99
LexA∆24aa (pH 4)	0.61 ± 0.003	1.84 ± 0.003	29.94 ± 0.13	0.98

**Figure 5:**
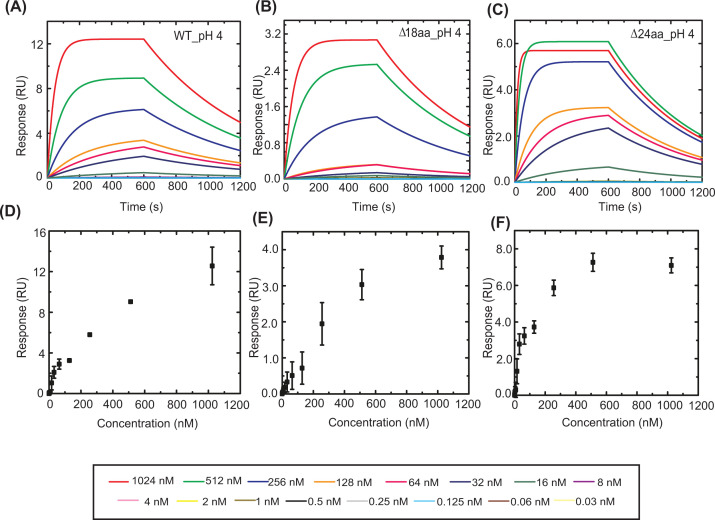
Kinetic analysis of DNA binding of Mtb LexA and its variants to *dnaE2* ‘SOS’ box at acidic pH Representative BLI sensograms showing the real-time concentration-dependent (each concentration depicted by a different color indicated in the box below) binding of WT Mtb LexA and its variants, LexAΔ18aa, and LexAΔ24aa to biotinylated 44 mer *dnaE2* ‘SOS’ box at pH 4 (**A**–**C**), the kinetic parameters obtained from which are tabulated in Table 2. The corresponding response versus concentration curves has been plotted in (**D**–**F**) from the results of three independent experiments.

We found that Mtb LexA exhibited maximal and optimal binding with the perfectly palindromic *dnaE2* ‘SOS’ box near physiological pH (pH 7.5). Surprisingly, Mtb LexA retained the ability to bind DNA even at pH 4, although with reduced affinity (Figure 5). The dissociation constants changed from 2.16 ± 0.01, 4.94 ± 0.03, and 34.4 ± 0.19 nM (at pH 7.5) to 75.99 ± 0.27, 29.94 ± 0.13, and 111 ± 0.33 nM (at pH 4) for WT, LexAΔ24aa, and LexAΔ18aa, respectively. Binding at acidic pH (pH 4) can thus be said to have reduced by nearly 35 times for the wild-type protein, nearly six times for LexAΔ24aa, and nearly 3.2 times for LexAΔ18aa when compared with physiological conditions (Table 2). From the kinetic parameters observed, we notice a significant reduction in association rate constants (*k*_on_) at low pH (pH 4) compared with physiological conditions (pH 7.5) for wild-type protein and its Δ24aa variant while the change is not so pronounced in the case of the Δ18aa variant. Dissociation rate constants (*k*_off_) in the case of WT Mtb LexA remained comparable in both the pH conditions tested but reduced by a power of 10 for the mutant proteins at low pH. Mutants seem to both associate and dissociate faster at physiological pH as compared with low pH and as for the wild-type protein, there has been a drastic reduction only in its rate of association to bind DNA at low pH conditions.

Our observations results corroborate with those made from *in vitro* studies that have assessed the effect of variations in pH in regulating the ‘SOS’ response for *E. coli* LexA [23–25]. Relan and colleagues reported that *E. coli* LexA bound to its operator maximally near physiological pH displaying about 10-fold better binding compared with that at pH 4 [23]. Similarly, we noticed a reduction in DNA binding by Mtb LexA as well, at acidic pH. It will be interesting to decipher the molecular events leading to this.

### DNA binding kinetics of Mtb LexA with different ‘SOS’ boxes

The differential gene expression profile following DNA damage led to the identification of genes that fall under direct regulation of LexA in Mtb [21]. However, DNA binding kinetics of LexA to ‘SOS’ boxes of these DNA damage-inducible genes remained uncharacterized. We, therefore, determined the DNA binding affinity for LexA and its mutants to different mycobacterial ‘SOS’ boxes (the kinetic parameters determined for the interaction of mutants to different ‘SOS’ boxes are provided in Supplementary Figure S4 and Table S3). The ‘SOS’ boxes chosen have unique characteristics (Table 1). While the *dnaE2* ‘SOS’ box is a perfect palindrome throughout (as mentioned in the previous section), *lexA* and *recA* ‘SOS’ boxes have one mismatch toward their 3′ ends (on the flank). *rv3074* ‘SOS’ box is unique in displaying a perfect palindrome of sequences on either side repeat flanks but showing mismatches in sequences between the flanks. All the genes whose ‘SOS’ boxes have been chosen for the present study are highly induced following DNA damage in Mtb [21].

**Figure 6:**
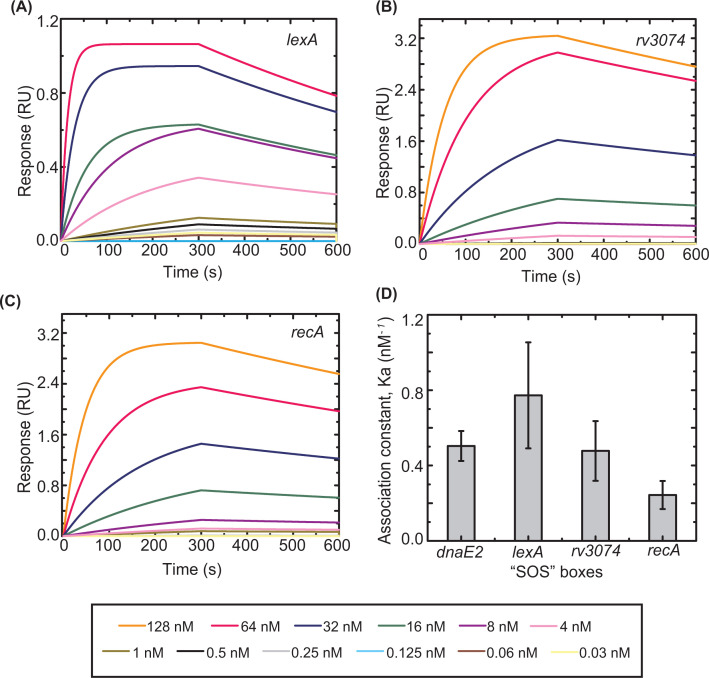
DNA binding kinetics of Mtb LexA to various ‘SOS’ boxes Representative BLI sensograms showing the real-time concentration-dependent (each concentration depicted by a different color indicated in the box below) binding of WT Mtb LexA to 44 mer biotinylated ‘SOS’ boxes of (**A**) *lexA*, (**B**) *rv3074*, and (**C**) *recA*, the kinetic parameters obtained from which are tabulated in Table 3. **(D**) Comparison in binding by plotting association constants (*K*a) of WT Mtb LexA to different ‘SOS’ boxes from three independent experiments is shown. Association constants (*K*a) are inverse of equilibrium dissociation constants or *K*_D_ values obtained from kinetic analyses.

Increasing concentrations of WT LexA and its variants (analytes) were allowed to interact with biotinylated ‘SOS’ boxes till saturation in binding was achieved. WT LexA was found to bind to different ‘SOS’ boxes with close affinities (Figure 6 and Table 3). *K*_D_ values ranged from 0.98 ± 0.01 nM for the *lexA* ‘SOS’ box to 3.86 ± 0.03 nM as noted for the *rv3074* ‘SOS’ box. The association rate was relatively higher for *dnaE2* and *lexA* ‘SOS’ boxes as compared with the other two ‘SOS’ boxes. The perfectly palindromic nature of the dnaE2 ‘SOS’ box facilitates faster association with LexA. *dnaE2* encodes an error-prone DNA polymerase; hence, its regulation must be strictly controlled. It is known that genes that are involved in mutagenesis such as these error-prone DNA polymerases are expressed later in the ‘SOS’ response cascade and are tightly controlled [26]. Our observation confirms the same in the case of Mtb.

**Table 3:** Kinetic parameters obtained from binding studies of WT Mtb LexA with various ‘SOS’ boxes performed using BLI

‘SOS’ boxes	*k*_on_ (M^−1^s^−1^) × 10^5^	*k*off (s^−1^) × 10^−3^	*K*_D_ (nM)	*R* ^2^
*dnaE2*	10.55 ± 0.06	2.28 ± 0.007	2.16 ± 0.01	0.99
*lexA*	10.27 ± 0.04	1.01 ± 0.004	0.98 ± 0.01	0.99
*rv3074*	1.39 ± 0.004	0.53 ± 0.004	3.86 ± 0.03	0.99
*recA*	1.61 ± 0.005	0.59 ± 0.004	3.63 ± 0.02	0.99

Although the association rate constants (*k*_on_) varied for different ‘SOS’ boxes, the corresponding dissociation rate constants (*k*_off_) are also seen to change proportionately; hence, the overall *K*_D_ is not widely altered for different ‘SOS’ boxes tested here. Though no drastic changes in binding affinity to different ‘SOS’ boxes are noted here, studying these repressor-DNA binding events extensively in the cellular context can reveal additional modes of regulation or factors modulating the expression patterns of ‘SOS’ responsive genes at the time of ‘SOS’ activation.

## Conclusion

Mtb LexA controls gene expression patterns of the crucial ‘SOS’ response pathway that facilitates mycobacterial adaptation to stress [12]. However, lack of thorough understanding at the molecular level, taking into account the unique regions of Mtb LexA that could potentially influence its interaction with DNA, prompted us to execute the present study by analyzing the impact of such truncations/ mutations on Mtb LexA–DNA interaction. Together, we present our detailed analysis of Mtb LexA and the role of its additional stretches of amino acids in regulating the ‘SOS’ response.

To begin with, the deletion and mutated variants displayed comparable secondary structure as that of wild-type Mtb LexA protein, inferred from the ellipticity measurements carried out using circular dichroism. Moreover, they retained the ability to form dimers as observed from size exclusion chromatography and crosslinking studies.

Qualitative estimation and comparative analysis of protein–nucleic acid interaction of the variants compared with the WT revealed that while the 24 amino acids extension at the N-terminal is not critical for Mtb LexA–DNA association, deletion of the 18 amino acids linker connecting the NTD and CTD of the protein resulted in a marked reduction in DNA binding compared with the full-length protein. The 18 amino acids present in the linker most likely accounts for the conformational flexibility of Mtb LexA to suitably bind DNA. Moreover, mutating the RKG motif in the DNA binding helix abolished LexA–DNA binding, highlighting the significance of strong evolutionary conservation of this motif across different organisms.

The quantitation of DNA binding in real-time has been carried out using BLI and kinetic parameters of Mtb LexA–DNA interaction have been determined. The binding affinity of WT Mtb LexA (*K*_D_ 2.16 ± 0.01 nM) and LexAΔ24aa (*K*_D_ 4.94 ± 0.03 nM) was within 2-fold range, while a 17-fold reduction with LexAΔ18aa (*K*_D_ 34.4 ± 0.19) and no observable binding with RKG/AAA mutant was observed. Mtb LexA was found to bind different ‘SOS’ boxes under mycobacterial ‘SOS’ regulation with comparable affinity. Association with perfectly palindromic sequence was found to be stronger. However, since association and dissociation rates changed proportionately for all the ‘SOS’ boxes, the overall affinities were found to fall in a close range. Although Mtb LexA binds to different ‘SOS’ boxes with comparable affinity *in vitro*, in the cellular context, the time and spatial regulation of ‘SOS’ genes might be altered by other transcription factors, intracellular pH, specific cations, and anions. *In vivo* studies may uncover their actual regulation under DNA damaging and normal conditions. DNA binding assays under both physiological and extreme acidic pH conditions *in vitro* revealed that mycobacterial LexA retains DNA binding even at pH as low as 4, albeit with reduced affinity as compared with its optimum binding at physiological pH. Taken together, our study provides a better understanding of the real-time kinetics of mycobacterial ‘SOS’ regulation. Extensive characterization of Mtb LexA in controlling one of the key stress-responsive pathways of the bacteria is imperative and will facilitate designing unconventional and yet more effective therapeutic strategies in counteracting TB infection.

## Supplementary Material

Supplementary Figures S1-S4 and Tables S1-S3

## Data Availability

All supporting data and sequence information are included within the main article and its supplementary material.

## Abbreviations

BLI: Bio-layer Interferometry
CTD: C-terminal domain
DBD: DNA-binding domain
NTD: N-terminal domain
PMSF: phenylmethylsulfonyl fluoride
