# Supplementary material for: Real-time kinetic studies of Mycobacterium tuberculosis LexA–DNA interaction
Source: Biosci Rep. 2021 Nov 18;41(11):BSR20211419. doi: 10.1042/BSR20211419 (PMC8607333; doi:10.1042/BSR20211419)
Supplement: Supplementary Figures S1-S4 and Tables S1-S3 [file BSR-2021-1419_supp.pdf]

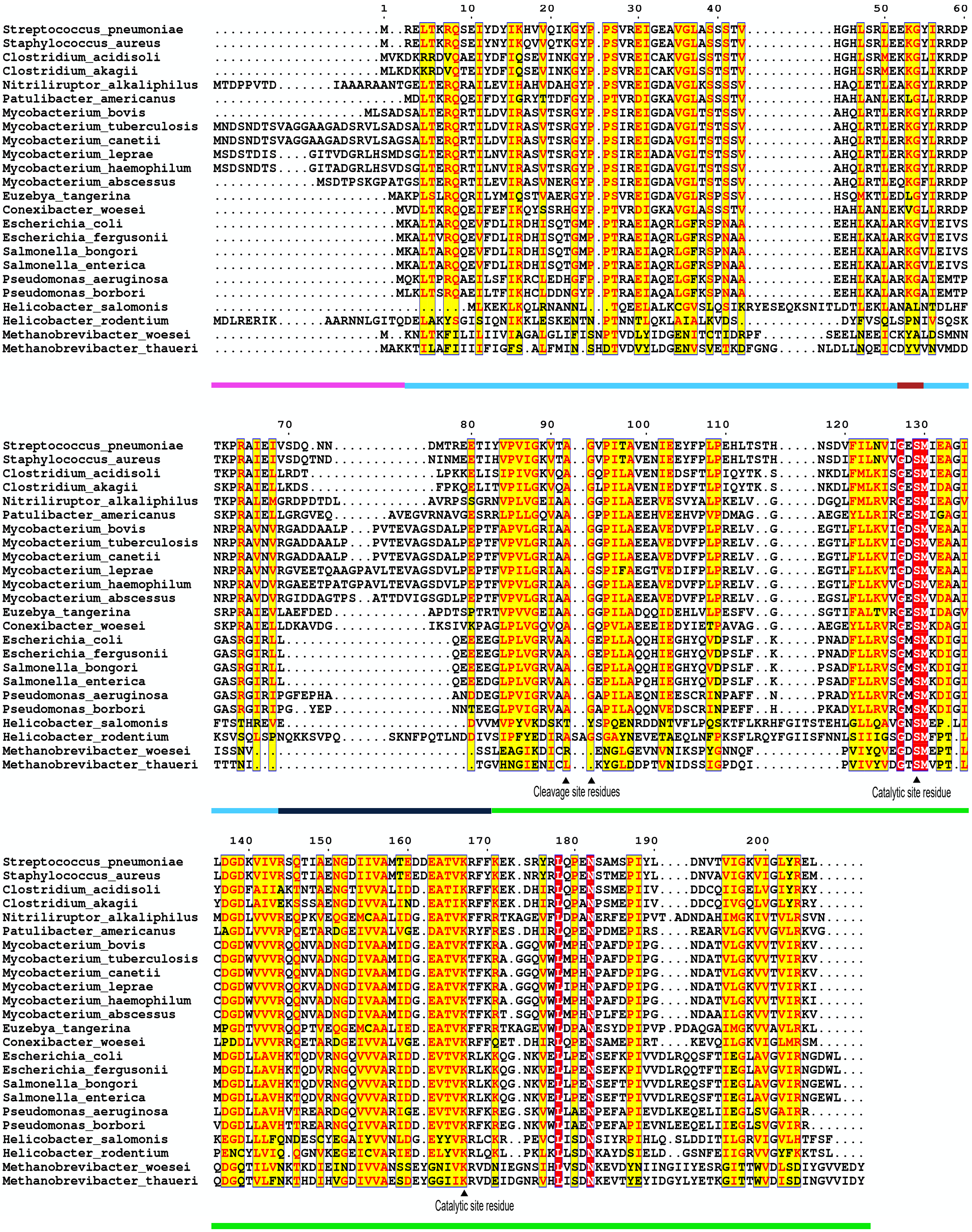
Streptococcus_pneumoniae
Staphylococcus_aureus
Clostridium_acidisoli
Clostridium_akagii
Nitriliruptor_alkaliphilus
Patulibacter_americanus
Mycobacterium_bovis
Mycobacterium_tuberculosis
Mycobacterium_canetii
Mycobacterium_leprae
Mycobacterium_haemophilum
Mycobacterium_abscessus
Euzebya_tangerina
Conexibacter_woesei
Escherichia_coli
Escherichia_fergusonii
Salmonella_bongori
Salmonella_enterica
Pseudomonas_aeruginosa
Pseudomonas_borbori
Helicobacter_salomonis
Helicobacter_rodentium
Methanobrevibacter_woesei
Methanobrevibacter_thaueri
Cleavage site residues
Catalytic site residue
Catalytic site residue

Figure S1. Multiple sequence analysis of LexA protein. Comparison between sequences of Mtb LexA with LexA proteins from representatives from Actinobacteria, Proteobacteria, Euryarchaeota, and Firmicutes are shown. The first 24 amino acids extension, the N-terminal DBD, linker region, and CTD are represented as colored bars below the sequences in magenta, light blue, dark blue, and light green respectively. Conserved DNA binding residues are shown with a brown bar representation below. Sequence alignment was done using Clustal Omega and ESPript was used to generate the figure.

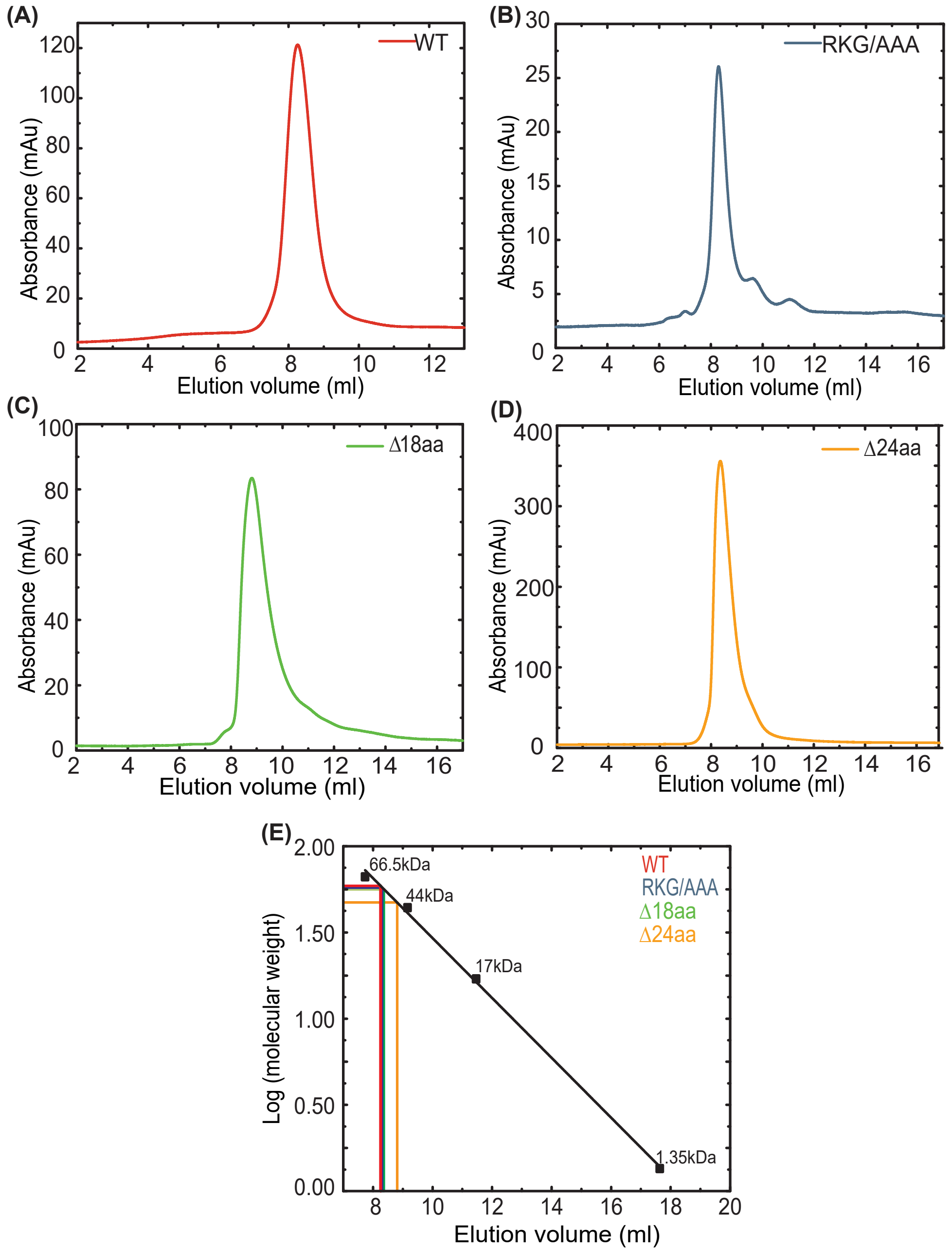

(A)
WT
Absorbance (mAu)
Elution volume (ml)
(B)
RKG/AAA
Absorbance (mAu)
Elution volume (ml)
(C)
Δ18aa
Absorbance (mAu)
Elution volume (ml)
(D)
Δ24aa
Absorbance (mAu)
Elution volume (ml)
(E)
66.5kDa
44kDa
17kDa
1.35kDa
WT
RKG/AAA
Δ18aa
Δ24aa
Log (molecular weight)
Elution volume (ml)

Figure S2. Analysis of the oligomeric state of Mtb LexA and its variants. Gel Filtration analysis of (**A**) WT Mtb LexA and its variants, (**B**) RKG/AAA, (**C**) LexAΔ18aa and (**D**) LexAΔ24aa. 500 µl was injected and run in Superdex 75 10/300 column pre-equilibrated with 20 mM Tris-Cl, pH 7.5, 100 mM NaCl, and 5% glycerol. (**E**) Approximate molecular weight of Mtb LexA and its variants plotted by comparing with standard markers.

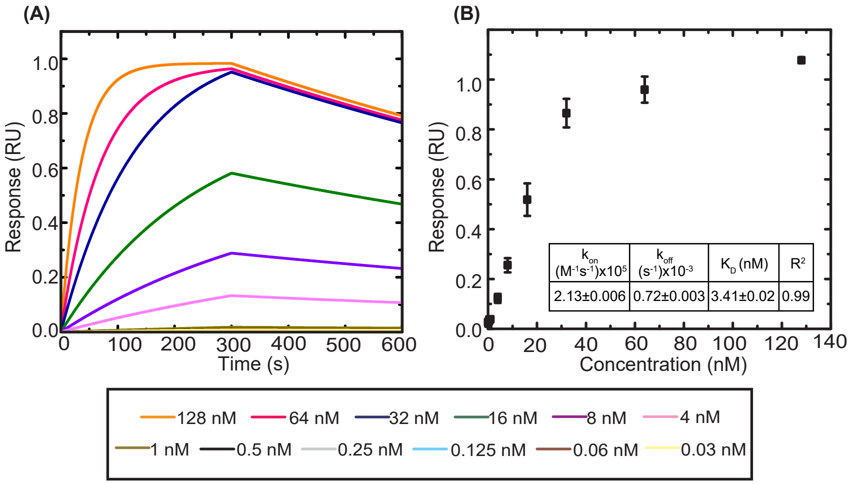

(A)
Response (RU)
1.0
0.8
0.6
0.4
0.2
0.0
0
100
200
300
400
500
600
Time (s)
(B)
Response (RU)
1.0
0.8
0.6
0.4
0.2
0.0
0
20
40
60
80
100
120
140
Concentration (nM)
k_on (M^-1s^-1)x10^5
k_off (s^-1)x10^-3
K_D (nM)
R^2
2.13±0.006
0.72±0.003
3.41±0.02
0.99
128 nM
64 nM
32 nM
16 nM
8 nM
4 nM
1 nM
0.5 nM
0.25 nM
0.125 nM
0.06 nM
0.03 nM

Figure S3. Assessment of DNA binding property of LexAΔ24aa (without N-terminal 6x His tag). Representative BLI sensogram showing the real-time concentration-dependent (each concentration depicted by a different color indicated in the box below) binding of LexAΔ24aa variant to biotinylated 44 mer lexA "SOS" box at physiological pH, the kinetic parameters obtained from which are tabulated and shown in the inset (**A**). The corresponding response versus concentration curve has been plotted in (**B**) from the results of three independent experiments.

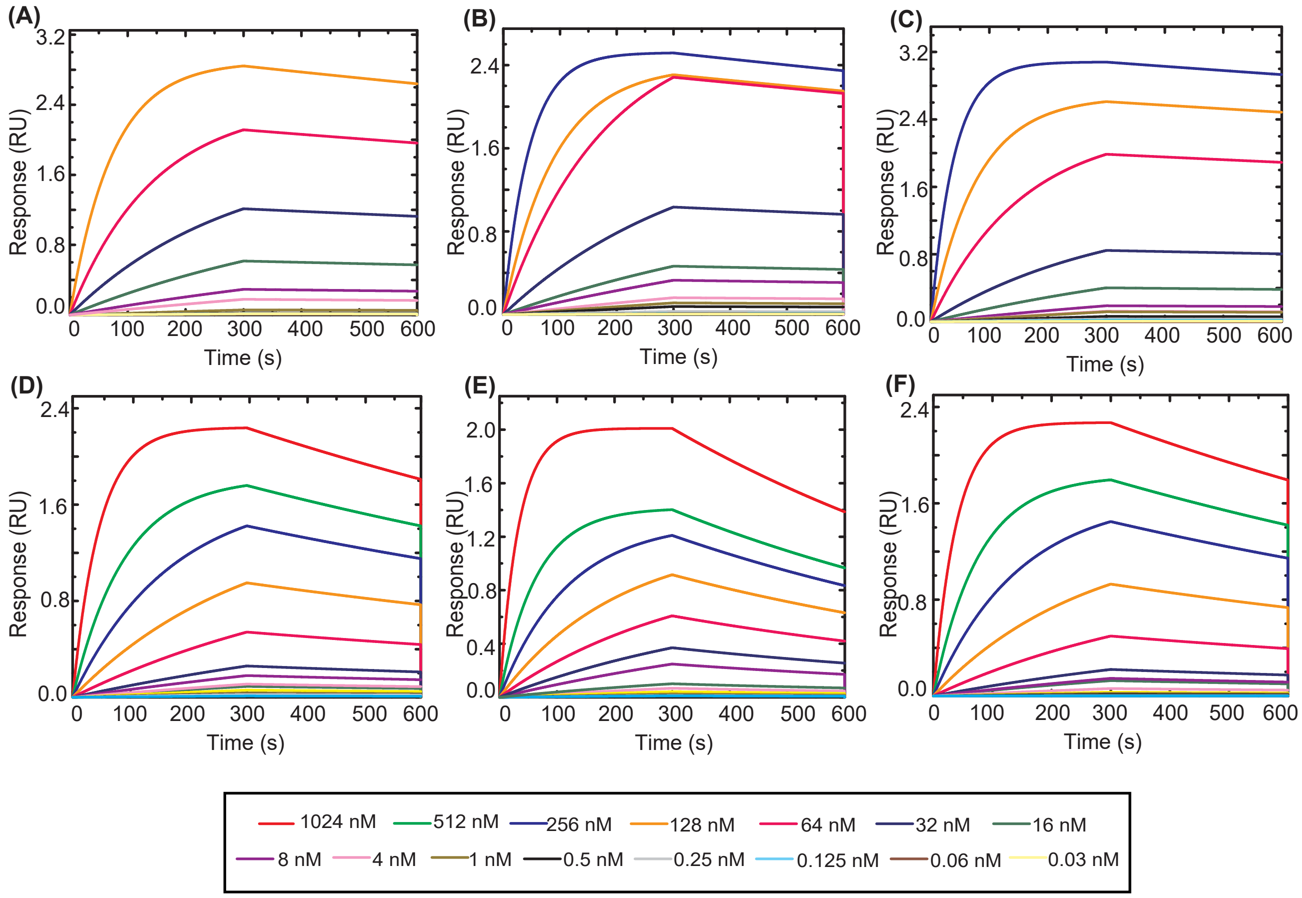
(A)
(B)
(C)
(D)
(E)
(F)
Response (RU)
Time (s)
1024 nM
512 nM
256 nM
128 nM
64 nM
32 nM
16 nM
8 nM
4 nM
1 nM
0.5 nM
0.25 nM
0.125 nM
0.06 nM
0.03 nM

Figure S4. Assessment of DNA binding properties of variants of Mtb LexA to various "SOS" boxes. BLI sensograms showing the real-time concentration-dependent (each concentration depicted by a different color indicated in the box below) binding of LexAΔ24aa to 44 mer biotinylated "SOS" boxes of (**A**) *lexA*, (**B**) *rv3074* and (**C**) *recA* and of LexAΔ18aa to 44 mer biotinylated "SOS" boxes of (**D**) *lexA*, (**E**) *rv3074* and (**F**) *recA*, respectively, the kinetic parameters obtained from which are tabulated in **Table S3**..

Table S1: Bacterial strains and plasmids

Name	Characteristics	Source
**Bacterial Strains**		
E.coli DH5α	F– φ80lacZΔ M15 Δ (*lacZYA-argF*) *U169 recA1 endA1 hsdR17* (rK– mK+) *phoA supE44* λ- *thi–1 gyrA96 relA1*	Laboratory stock
E.coli BL21(DE3)	F– *omp*T *hsdSB* (rB–, mB–) *gal dcm* (DE3)	Laboratory stock
**Plasmids**		
pET28a(+)	Expression vector for low copy number (pBR322 ori), strong phage promoter (T7), IPTG induction (lac operon), and Kanamycin selection (Kan^r)	Novagen
pET22b(+)	Expression vector (pBR322 ori), strong phage promoter (T7), IPTG induction (lac operon), and Ampicillin selection (Amp^r)	Novagen

Table S2: Oligonucleotide primers used in this study

Construct/Mutant	Forward Primer (FP) / Reverse Primer (RP) Sequences with restriction sites and base changes for mutation highlighted in bold
WT Mtb LexA	FP- 5´ ATGCCG **CATATG** ATGAACGACAGCAACGAC 3´
	RP- 5´ ATGCCG **GGATCC** TCAGACCTTGCGGATCAC 3´
Mtb LexAΔ18aa	FP- 5´ GTCAATGTGCGCGGTCCGGAACCCACCTTT 3´
	RP- 5´ AAAGGTGGGTTCCGGACCGCGCACATTGAC 3´
Mtb LexAΔ24aa	FP- 5´ ATAT **CATATG** TCGGCGCTGACCGAGCGGCAA 3´
	RP- 5´ ATGCCG **GGATCC** TCAGACCTTGCGGATCAC 3´
	RP- 5´ CCC **AAGCTT** GACCTTGCGGATCACCGT 3´
Mtb LexA RKG/AAA	FP- 5´ CTGCGCACCCTGGAG**GCGGCGGCG**TACCTACGCCGTGAC 3´
	RP- 5´ TCACGGCGTAGGTACGCCGCCGCCTCCAGGGTGCGCAG 3´

Table S3: Assessment of DNA binding properties of Mtb LexA variants to various "SOS" boxes: Kinetic parameters obtained from binding studies performed using BLI.

**Protein**	**"SOS" boxes**	**k_{on} ($M^{-1}s^{-1}$) x 10^5**	**k_{off} (s^{-1}) x 10^{-3}**	**K_D (nM)**	**R^2**
Mtb LexAΔ18aa	*dnaE2*	0.22±0.0007	0.76±0.004	34.40±0.19	0.99
	lexA	0.21±0.0008	0.71±0.004	34.53±0.25	0.99
	rv3074	0.28±0.0011	1.24±0.005	43.50±0.24	0.99
	recA	0.24±0.0009	0.79±0.004	32.55±0.22	0.99
Mtb LexAΔ24aa	*dnaE2*	1.06±0.003	0.52±0.003	4.94±0.03	0.99
	lexA	1.05±0.002	0.25±0.002	2.36±0.02	0.99
	rv3074	0.85±0.002	0.24±0.003	2.75±0.04	0.99
	recA	0.99±0.002	0.17±0.002	1.67±0.02	0.99
